# Biochemical and Molecular Investigation of In Vitro Antioxidant and Anticancer Activity Spectrum of Crude Extracts of Willow Leaves *Salix safsaf*

**DOI:** 10.3390/plants9101295

**Published:** 2020-09-30

**Authors:** Mourad A. M. Aboul-Soud, Abdelkader E. Ashour, Jonathan K. Challis, Atallah F. Ahmed, Ashok Kumar, Amr Nassrallah, Tariq A. Alahmari, Quaiser Saquib, Maqsood A. Siddiqui, Yazeed Al-Sheikh, Hany A. El-Shemy, Ahmed M. Aboul-Enein, Khalid M. Alghamdi, Paul D. Jones, John P. Giesy

**Affiliations:** 1 Chair of Medical and Molecular Genetics Research, Department of Clinical Laboratory Sciences, College of Applied Medical Sciences, King Saud University, P.O. Box 10219, Riyadh 11433, Saudi Arabia; tariqs@live.com (T.A.A.); yalsheikh@ksu.edu.sa (Y.A.-S.); 2 Department of Basic Medical Sciences, Kulliyyah (College) of Medicine, International Islamic University Malaysia, Kuantan 25200, Pahang, Malaysia; 3 Toxicology Centre, University of Saskatchewan, Saskatoon, SK S7N 5B3, Canada; j.challis@usask.ca (J.K.C.); pdj055@mail.usask.ca (P.D.J.); jgiesy@aol.com (J.P.G.); 4 Department of Pharmacognosy, College of Pharmacy, King Saud University, Riyadh 11451, Saudi Arabia; afahmed@ksu.edu.sa; 5 Department of Pharmacognosy, Faculty of Pharmacy, Mansoura University, Mansoura 35516, Egypt; 6 Vitiligo Research Chair, College of Medicine, King Saud University, Riyadh 11451, Saudi Arabia; aknirankari@gmail.com (A.K.); kmgderm@gmail.com (K.M.A.); 7 Biochemistry Department, Faculty of Agriculture, Cairo University, Giza 12613, Egypt; amotagly@cu.edu.eg (A.N.); helshemy@yahoo.com (H.A.E.-S.); aboul.enein1@gmail.com (A.M.A.-E.); 8 Zoology Department, College of Sciences, King Saud University, P.O. Box 2455, Riyadh 11451, Saudi Arabia; quaiser.saquib0@gmail.com (Q.S.); tellmaqsood@rediffmail.com (M.A.S.); 9 Department of Dermatology, College of Medicine, P.O. Box 240997, King Saud University, Riyadh 11322, Saudi Arabia; 10 School of Environment and Sustainability, University of Saskatchewan, Saskatoon, SK S7N 5B3, Canada; 11 Department of Veterinary Biomedical Sciences, University of Saskatchewan, Saskatoon, SK S7N 5B4, Canada; 12 Department of Environmental Science, Baylor University, Waco, TX 76798-7266 (254), USA

**Keywords:** *Salix safsaf*, cytotoxicity, polyphenols, flavonoids, mass spectrometry, apoptosis, natural products

## Abstract

Organic fractions and extracts of willow (*Salix safsaf*) leaves, produced by sequential solvent extraction as well as infusion and decoction, exhibited anticancer potencies in four cancerous cell lines, including breast (MCF-7), colorectal (HCT-116), cervical (HeLa) and liver (HepG2). Results of the MTT assay revealed that chloroform (CHCl_3_) and ethyl acetate (EtOAc)-soluble fractions exhibited specific anticancer activities as marginal toxicities were observed against two non-cancerous control cell lines (BJ-1 and MCF-12). Ultra-high-resolution mass spectrometry Q-Exactive™ HF Hybrid Quadrupole-Orbitrap™ coupled with liquid chromatography (UHPLC) indicated that both extracts are enriched in features belonging to major phenolic and purine derivatives. Fluorescence-activated cell sorter analysis (FACS), employing annexin V-FITC/PI double staining indicated that the observed cytotoxic potency was mediated via apoptosis. FACS analysis, monitoring the increase in fluorescence signal, associated with oxidation of DCFH to DCF, indicated that the mechanism of apoptosis is independent of reactive oxygen species (ROS). Results of immunoblotting and RT-qPCR assays showed that treatment with organic fractions under investigation resulted in significant up-regulation of pro-apoptotic protein and mRNA markers for Caspase-3, p53 and Bax, whereas it resulted in a significant reduction in amounts of both protein and mRNA of the anti-apoptotic marker Bcl-2. FACS analysis also indicated that pre-treatment and co-treatment of human amniotic epithelial (WISH) cells exposed to the ROS H_2_O_2_ with EtOAc fraction provide a cytoprotective and antioxidant capacity against generated oxidative stress. In conclusion, our findings highlight the importance of natural phenolic and flavonoid compounds with unparalleled and unique antioxidant and anticancer properties.

## 1. Introduction

Despite significant progress made towards discovery of potent chemotherapeutic agents, cancer remains an aggressive and devastating disease. Worldwide, cancer is the second-leading cause of mortalities, after cardiovascular diseases, with a striking reported incidence of 18.1 million new cases and 9.6 million deaths in 2018 [1]. Cancer is now ranked as the first cause of mortalities in 21 states of the United States of America, with estimated new cases and fatalities in 2018 of around 1.7 million and 600,000, respectively [2]. In 2010, cancer was estimated to have an astonishing total annual economic burden of approximately USD 1.16 trillion [3]. Currently, therapeutic protocols used to treat cancers rely on both traditional treatments, including surgery, chemotherapy and radiotherapy and complementary and alternative medicine (CAM) strategies, utilizing natural products (NPs). Over the past 60 years, more than 200 anticancer molecules have been approved of which 50% are NPs [4,5]. Anticancer activities of several novel NP and NP-derived drug pharmacophores have been tested at both pre-clinical and clinical (Phases I, II and III) stages, offering a bright prospect for future registration and approval of several of them. However, many of these candidate drugs have not made it to clinical trials, or their clinical trials have been halted or discontinued. This calls for better-coordinated efforts to identify novel bioactive pharmacophores leading to their advancement through pre-clinical investigation into clinical trials [5]. Historically, traditional herbal medicine has been intensively employed by indigenous cultures around the globe to combat a myriad of pathological conditions; approximately 60% and 80% of the population of the world and developing countries, respectively, rely on herbal medicine [6]. Plant-derived NPs from roots, bark and leaves of terrestrial plants are fundamental to CAM-based cancer therapeutic strategies in several countries. More than 3000 medicinal plants have been identified to possess antineoplastic activities [7], and thirty plant-derived NPs have been tested against cancer in clinical trials [8]. Examples of some NPs derived from plants that have been employed as anticancer chemotherapeutic agents include vinblastine, camptothecin, podophyllotoxin, paclitaxel (Taxol^®^), topotecan (Hycamtin^®^), etoposide phosphate (Etopophos^®^) and homoharringtonine (Synribo^®^) [4].

Despite the significant cost of discovery and development of novel, synthetic, chemotherapeutic drugs, their repertoire that is currently employed in clinical settings has failed to fulfill expectations over the past decade [9]. This is attributed, in part, to intrinsic non-target toxicity to normal cells impacting their regulatory functions, which justifies the urgent and increasing demand to develop new, effective and inexpensive anticancer drugs from alternative sources with safer and minimal side effects [9]. Compared to their synthetic counterparts, compounds derived from plants exhibit lesser toxic potencies to normal cells, with alternative cell-death promoting mechanisms [10]. Mixtures of phytochemicals, found in a varied and balanced diet, have been reported to exert synergistic effects leading to enhanced anticancer bioactivity and health benefits that cannot be paralleled by ingestion of single active compounds [11,12]. This has led to an unprecedented interest by researchers in the investigation of plants as rich sources of bioactive anticancer compounds [8]. Substantial research efforts are required to identify NP-producing plants as potential anticancer agents, employing them as alternative natural therapeutics that efficiently target tumors.

The genus *Salix*, commonly known as willow, includes almost 350 species. *Salix* species are cultivated in countries with temperate and semi-tropical climates as in the Middle Eastern country Egypt. In the Ebers papyrus, ancient Egyptians reported that willow could be used as an analgesic [13]. Willow bark has been reported to be rich in salicin, which is an aryl-β-D-glucoside, a natural precursor for acetyl-salicylic acid, which is the active ingredient in the analgesic pharmaceutical Aspirin^®^. Supplements derived from bark extracts of several species of willow are widely commercialized for their antipyretic, analgesic and anti-inflammatory properties [13]. A decoction of willow (*Salix safsaf* L., Salicaceae) leaf has been used in traditional treatments including antileukemic applications in the Egyptian countryside [1]. Our research group was the first to report that aqueous and ethanolic extracts exhibit potent salicin-associated cytotoxic properties against acute myeloid leukemia (AML) cells [14]. Moreover, we have investigated the anti-leukemic effects of aqueous extract against three types of leukemia cell types both in vitro and in vivo in mice. This observed anti-leukemic effect has been suggested to take place via a DNA damage-induced apoptotic mechanism [15]. However, the full scope of the extract-mediated cytotoxicity spectrum against other types of cancer cells remained undetermined. Additionally, these reports have not provided detailed investigations of specific constituents of willow extracts or their modes-of-action, such that current knowledge of the molecular mechanisms underlying this activity remained largely unknown. 

The three-fold primary objectives of this study were to: (i) use sequential extraction procedures with organic solvents of increasing polarity to prepare organic-soluble fractions containing potentially bioactive compounds, as well as infusion and decoction extracts; (ii) employ a bioassay-directed approach to screen for the in vitro anti-proliferative activity of willow fractions/extracts against four different human carcinoma cell line models, including breast (MCF-7), colon (HCT-116), cervix (HeLa) and liver (HepG2); (iii) elucidate molecular mechanisms of bioactivity of willow fractions/extracts by profiling relative expressions of proteins and mRNA of selected markers of pro- and anti-apoptotic responses and by studying cell cycle progression using reverse transcription-quantitative polymerase chain reaction (RT-qPCR), immunoblotting and flow cytometry techniques, respectively. 

## 2. Materials and Methods

### 2.1. Ethical Approval

The study was ethically approved by the Institutional Review Board of the Health Sciences Colleges Research on Human Subjects, King Saud University College of Medicine (E-20-4585) on 12 February 2020.

### 2.2. Chemicals and Supplies

All organic solvents were analytical grade reagents (AR) procured from Merck Chemical Inc. (Darmstadt, Germany). MTT (3-[4,5-dimethylthiazol-2yl]-2.5-diphenylterazolium bromide) was obtained from Sigma Aldrich company (St Louis, MO, USA). Fetal bovine serum (FBS), Dulbecco’s Modified Eagle Medium (DMEM)/high glucose, L-glutamine and penicillin/streptomycin were purchased from Gibco Inc. (NY, USA). Total RNA extraction, cDNA synthesis, reverse transcription-quantitative polymerase chain reaction (RT-qPCR) kits, reagents and PCR oligo primers were ordered and purchased from Qiagen (Hilden, Germany). Corning^®^ 96-well clear flat-bottom polystyrene TC-treated Microplates, Corning^®^ 75 cm^2^ cell culture flasks with vent cap, Falcon^®^ (15 and 50 mL) polystyrene centrifuge tubes and sterile individually-wrapped Stripette™ serological polystyrene pipettes were purchased from Corning^®^ USA. All protein chemistry reagents and buffers were obtained from Bio-Rad Laboratories GmbH (Munich, Germany).

### 2.3. Sample Collection and Preparation

Taxonomically-authenticated willow leaves (*Salix safsaf* L.) were collected from the countryside in the outskirts of Giza, Egypt [14,15]. *S. salix* leaves were identified by Prof. Ahmed Aboul-Enein, Faculty of Agriculture, Cairo University, Egypt. A voucher sample (Code no. EgP01-2018) was deposited at the Department of Pharmacognosy, College of Pharmacy, King Saud University, with Prof. Atallah Ahmed. The focus was on harvesting young emerging leaves since they exhibit the most significant metabolic activity and higher production of secondary metabolite concentrations, including potentially bioactive compounds [14,15]. Freshly harvested leaves were placed on trays and visually inspected for wilting or microbial contamination; only healthy leaves were subjected to further analysis. Any contaminating debris was removed, and leaves were subjected to three consecutive rinsing steps with tap water, sterilized water and finally distilled water. Cleaned, healthy and fresh leaves were air-dried at room temperature (RT) for 7 days in the laboratory, out of direct sun light. Next, air-dried leaves were finely powdered by use of a handheld electric coffee grinder and subsequently stored until further use at 4 °C in a tightly-sealed light-poof stainless steel container. A 1 g silica gel sachet was added as a desiccant to prevent humidity. 

### 2.4. Preparation of Crude Fractions/Extracts of Salix safsaf Leaves

**Figure 1:**
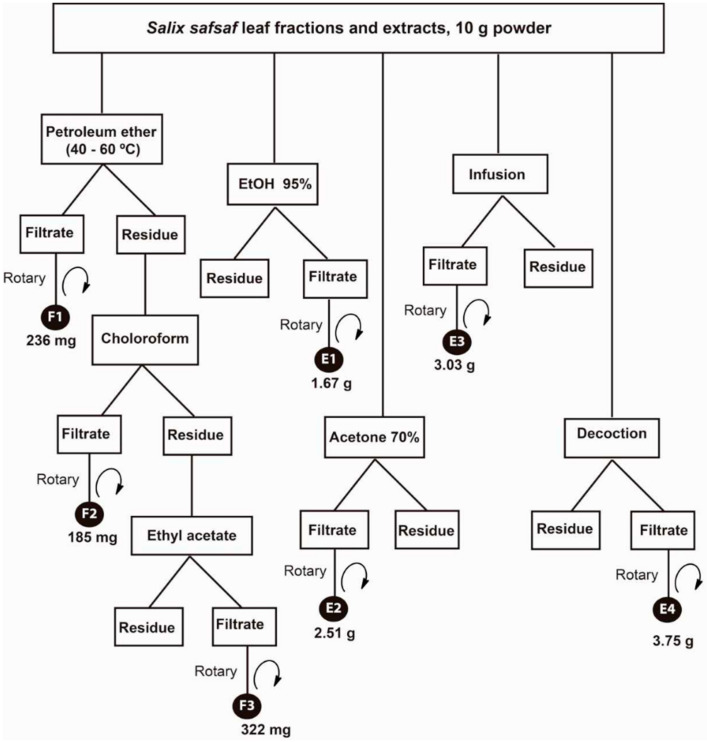
Scheme of the organic solvent fractionation and extraction and strategy employed to yield *Salix safsaf* leaf fractions/extracts. Fractions F1–F3 were those extracted by petroleum ether (**F1**, 236 mg), chloroform-CHCl_3_ (**F2**, 185 mg), and ethyle acetate-EtOAc (**F3**, 322 mg), sequentially. Extracts E1–E4 were those extracted by ethanol (**E1**, 1.67 g), hydro-acetone (**E2**, 2.51 g), aqueous infusion (**E3**, 3.03 g) and aqueous decoction (**E4**, 3.75 g).

A sequential extraction procedure was employed with three consecutive organic solvents (light petroleum ether, chloroform and ethyl acetate-alcohol mixture), followed by three independent extraction steps with 95% EtOH; and aqueous infusion and decoction that produced a total of seven fractions and extracts (Figure 1).

Briefly, a sample of the powdered air-dried willow (*S. safsaf*) leaves (10 g) was extracted sequentially with light petroleum ether (40–60 °C), CHCl_3_ and EtOAc (250 mL × 2 times, each) by use of sonication for 30 min at RT. Supernatants were collected by centrifugation at 6000× *g* for 15 min, followed by evaporation under reduced pressure at 40 °C to yield solvent-free petroleum ether (**F1**, 236 mg), CHCl_3_ (**F2**, 185 mg), and EtOAc (**F3**, 322 mg) fractions, respectively. In parallel, two powdered leaf samples (10 g, each) were extracted with 95% EtOH and 70% hydro-acetone, separately, in the same way as mentioned above to yield EtOH extract (**E1**, 1.67 g) and hydro-acetone extract (**E2**, 2.51 g). Another two powdered samples (10 g, each) were separately infused (at 95 °C/10 min) and boiled (10 min) in distilled water (250 mL × 2 times, each). After cooling, each mixture was decanted by centrifugation and evaporated by lyophilization to produce the dried aqueous extracts (**E3**, 3.03 g and **E4**, 3.75 g), respectively. Dried extracts were stored at 4 °C. For bioassays, stock solutions of each extract (1 mg/mL) were prepared in dimethyl sulfoxide (DMSO) and frozen at −20 °C until further use.

### 2.5. Cell Lines and Culturing Conditions

All cell lines utilized in this study were initially obtained from the American Type Culture Collection (ATCC, Manassas, VA, USA) and were maintained in freezing/storage medium containing 90% fetal bovine serum (FBS)/10% DMSO at −150 °C. Four human cancerous cell lines were used in this study, namely the cervical adenocarcinoma (HeLa, ATCC^®^ CCL-2™), colorectal adenocarcinoma (HCT-116, ATCC^®^ CCL-247™), mammary adenocarcinoma (MCF-7, ATCC^®^ HTB-22™) and hepatocellular carcinoma (HepG2, ATCC^®^ HB-8065™). For comparison of cytotoxicity, the noncancerous skin fibroblast BJ-1 (ATCC^®^ CRL-2522™) and epithelial breast MCF-12 (ATCC^®^ CRL-10782™) cell lines were used. Human amniotic epithelial (WISH, (ATCC^®^ CCL-25™) was used for measuring the cytoprotective activity against oxidative stress. Cell lines were cultured in DMEM/high glucose supplemented with 2 mM L-glutamine, 10% FBS and 1% penicillin/streptomycin. Then, sub-confluent cultures (80–90%) were trypsinized (Trypsin 0.05%/0.53 mM EDTA) and spilt depending on the seeding ratio recommended by ATCC [16,17].

### 2.6. Screening for Antiproliferative Activity by MTT Assay

Cytotoxic effects of crude willow fractions and extracts on viabilities of cancerous cells were investigated by assessing the ability of reducing enzymes present in viable cells to transform MTT into formazan crystals according to our previous reports [16,17]. Seven *Salix safsaf* fractions/extracts were initially screened for anti-proliferative activity at one concentration (50 μg/mL) against the above-mentioned cell lines (one-dose pre-screening). Briefly, cells cultured in complete medium were seeded into 96-well microtiter plates (in octuplicates) with 2 × 10^4^ cells per well and incubated under a humidified atmosphere of 5% CO_2_ at 37 °C for 24 h. The cell medium in test wells was then changed to cell medium containing only 5% FBS (5% medium). Test wells contained the seven willow fractions/extracts at a concentration of 50 μg/mL. At the same time, control wells contained an equivalent volume of the vehicle (DMSO). After incubation at 37 °C for 72 h, 5% medium in control and test wells were replaced by 100 µL/well of MTT (0.5 mg/mL) in phosphate-buffered saline (PBS) and incubated at 37 °C for additional 3 h. MTT solution were removed and the purple formazan crystals formed at the bottom of the wells were dissolved using 100 μL isopropyl alcohol/well with shaking for 2 h at room temperature. The absorbance at 549 nm was read on a microplate reader (ELX 800; Bio-Tek Instruments, Winooski, VT, USA). The dose–response curves of the two most effective fractions/extracts (F2 and F3) in one-dose pre-screening for each cell line were established with escalating concentrations of 6.25, 12.5, 25, 50, 100 and 200 µg/mL. Concentrations causing 50% inhibition of growth of cells (IC_50_) were calculated. The cytotoxic activity of the anticancer drug dasatinib, a potent, multi-targeted kinase inhibitor of BCR-ABL and SRC family kinases [18], against the above-mentioned cell lines was measured at the same concentrations of tested compounds and utilized as a standard for comparative purposes (data not shown).

### 2.7. Apoptosis and Cell Cycle Analysis by Flow Cytometry

Early changes in cell surfaces, associated with apoptosis and cell cycle arrest of cancer cells, treated with willow organic-soluble fractions were investigated by use of flow cytometry with the FITC-Annexin V/Propidium Iodide (PI) Apoptosis Detection Kit I (BD Biosciences Company, NJ, USA) by use of fluorescence-activated cell sorter analysis (FACS) Canto II flow cytometry system as previously described by us [19]. MCF-7 cells were treated with IC_50_ concentrations of F2 (128.1 µg/mL) and F3 (111.72 µg/mL) for 72 h. F2 and F3 were chosen as they exhibited the most potent anti-proliferation potential out of all the seven prepared *S. salix* fractions and extracts. In order to set up the compensation and quadrant parameters, an unstained reference population of cells was employed, whereas the untreated control cell population was utilized to define the basal level of apoptotic and dead cells. BD Diva software version 6.0 was used for flow cytometric analysis. To determine the role of oxidative stress and intracellular reactive oxygen species (ROS) generation in apoptosis of cells, caused by exposure to fractions of willow leaves, the intracellular peroxide-dependent oxidation of 2′,7′-dichlorodihydrofluoresceindiacetate (DCFH-DA) into a fluorescent compound, 2′,7′-dichlorofluorescein (DCF) was carried out as previously described [17]. The willow EtOAc-soluble fraction (F3) was specifically chosen as it exhibited the most potent anti-proliferative activity against MCF-7 cells (Table 1). In brief, MCF-7 cells were treated by the 100, 400 and 800 µg/mL concentrations of F3 for 72 h at 37 °C in 5% CO_2_ atmosphere. Then, cells were washed and stained appropriately by 5 μM of DCFH-DA at 37 °C for 10 min. Cell detection at multiple wavelengths (485 and 530 nm) was conducted using a fluorescence microscope (Nikon, Eclipse E600). WISH cells were utilized for the study of cell cycle progression and cyto-protective effects of F3 against hydrogen peroxide (H_2_O_2_) exposure according to our previously published protocol [20]. The design employed WISH cells treated with 0.1% DMSO as a solvent control, 1 mM H_2_O_2_ as positive control and co-treated with F3 (100, 400 and 800 µg/mL) plus 1 mM H_2_O_2_ for 24 h. Cells were harvested and centrifuged at 3600× *g* for 5 min. Pellets were resuspended in 500 µL of PBS. Cells were fixed with equal volume of chilled 70% ice-cold ethanol and incubated at 4 °C for 1 h. After two successive washes with PBS at 3600× *g* for 5 min, cell pellets were resuspended in PBS and stained with 50 µg PI/mL containing 0.1% Triton X-100 and 0.5 mg/mL RNAase A for 1 h at 30 °C in the dark. Fluorescence of the PI was measured by FACS by use of a Beckman Coulter flow cytometer (Coulter Epics XL/Xl-MCL, Miami, USA) through a FL-4 filter (675 nm) and 10,000 events were acquired as previously reported by us [21]. Data were analyzed by Coulter Epics XL/XL-MCL, System II Software, Version 3.0. Cell debris was characterized by a low FSC/SSC and was excluded from the analysis.

### 2.8. Gene Expression Profiling by RT-qPCR

Analyses of mRNA transcripts of key pro- and anti-apoptotic marker genes were conducted with MCF-7 cells exposed to 100, 400 or 800 µg/mL doses of F3 for 72 h [16]. Briefly, RNA was isolated by use of the Total RNA Purification Kit (Norgen Biotek Corp., Thorold, ON, Canada) according to the instructions of the manufacturer. Assessments of RNA quality, genomic DNA elimination and cDNA synthesis were carried out as previously described [16]. Amplification programs and PCR amplicon specificity were performed and assessed by use of a Rotor-Gene Q 5-Plex HRM thermal cycler (Qiagen, Germany) with QuantiTect SYBR-Green PCR Kit (Qiagen, Germany) as previously documented following standard protocols [16]. The following primers were used: *Hs_P53_1_SG QuantiTect Primer Assay (QT00060235); Hs_CASP3_1_SG QuantiTect Primer Assay (QT00023947)*; *B-cell lymphoma 2 Hs_BCL2_1_SG QuantiTect Primer Assay (QT00025011); Bcl-2-like protein Hs_BAX_1_SG QuantiTect Primer Assay (QT00031192)*; and *18S* rDNA house-keeping (HK) gene *Hs_RRN18S_1_SG QuantiTect Primer Assay (QT00199367)*. PCR thermal cycling program and gene expression analysis to determine the fold-change relative to the *18S* gene were essentially performed as previously reported [16].

### 2.9. Extraction of Protein and Western Blot Analysis

Western blot analyses were conducted as previously described [19]. Collected cells were frozen and then homogenized in extraction buffer containing 150 mM NaCl, 50 mM Tris-HCl pH 7.5, 10 mM MgCl_2_, 1 mM PMSF, 0.1% NP-40 and 1× complete protease inhibitor (Roche), by use of a stick “pellet pestle blue” (Sigma). Extracts were kept on ice and clarified by centrifugation at 13,000× *g* for 10 min at 4 °C. Supernatants were carefully collected into a new tube and centrifuged again for 10 min at 13,000× *g* to remove all plant debris. This second supernatant was transferred to a new tube and the protein content was quantified by the Bradford protein assay method (Bio-Rad^®^). Sodium dodecyl sulphate-polyacrylamide gel electrophoresis (SDS-PAGE) and immunodetection of selected pro-apoptotic (p53, Bax, Caspase-3) and anti-apoptotic (Bcl-2) protein markers were essentially conducted as detailed in our recently published protocol [19].

### 2.10. Orbitrap Mass Spectrometry

Stock solutions were made at approximately 250 mg/L by dissolving 2.5 mg of CHCl_3_ (F2) or EtOAc (F3) soluble fractions into 10 mL of HPLC grade methanol (Fisher Scientific, Mississauga, ON, Canada). Dilutions to 10 and 100 mg/L solutions in 50:50 water:methanol were used for instrumental analysis. Analyses were conducted using a Vanquish UHPLC and Q-Exactive™ HF Quadrupole-Orbitrap™ mass spectrometer (Thermo-Fisher, Mississauga, ON, Canada). LC separation was achieved with a Kinetex 1.7 µm C18 LC column (100 × 2.1 mm) (Phenomenex, Torrance, CA, USA) by gradient elution with 95%:5% water:methanol (A) and 100% methanol (B), unbuffered for negative mode and containing 0.1% formic acid for positive mode. A solvent flow rate of 0.1 mL/min and column temperature of 40 °C were used. The gradient elution began at 5% B and ramped to 100% B linearly over 20 min, holding at 100% B for 7 min, before re-equilibrating to 5% B for 8 min over a total run time of 35 min. Samples were ionized by heated electrospray ionization (HESI) with the following source parameters for positive/negative mode: sheath gas flow = 35/30; aux gas flow = 10/8; sweep gas flow = 1; aux gas heater = 400/300 °C; spray voltage = 3.8/2.7 kV; S-lens RF = 60; capillary temperature = 350 °C. A Full MS method was used with the following scan settings: 120,000 resolution, AGC target = 1 × 10^6^, max injection time = 100 ms, and a full MS scan range of 100–1000 m/z.

Compound Discoverer 2.1 SP1 (Thermo Fisher) was used to identify unknown peaks and generate elemental compositions based on exact mass data with a 1 ppm mass tolerance. The built-in workflow template used was Untargeted research workflow without statistics to find and identify unknown compounds which perform retention time alignment, unknown compound detection, and compound grouping across all samples. Elemental compositions for all compounds are predicted, chemical background is hidden using blank samples, and compound structures can be identified through ChemSpider (exact mass or formula). In positive and negative mode 2560 and 973 unique features were identified. The following post-analysis filtering was conducted to reduce the number of features: 1) only peaks appearing in sample extracts and not in blanks were considered; 2) minimum peak areas were ≥1 × 10^5^; and 3) only identified peaks with area ratios (10 ppm/100 ppm) falling between 0.08 and 0.12 were considered.

### 2.11. Statistical Analyses

Statistical analyses were conducted by use of Statistical Package for Social Sciences (SPSS; IL, USA) for Windows version 17.0. Assumptions of normality and homogeneity of variance were evaluated using the Shapiro–Wilks and Levene’s tests, respectively. Since data did not always meet these assumptions, the non-parametric, Man–Whitney U test was employed to identify significant differences among treatments with fractions/extracts and vehicle solvent control-treated cells at equivalent dilutions. Data are represented as mean ± standard deviation (SD) with 3 individual experiments each in duplicate. Treatments were considered statistically significant at *p* < 0.05.

## 3. Results

### 3.1. Effect of the Different Fractions and Extracts on the Proliferation of Cancerous Cells

**Figure 2:**
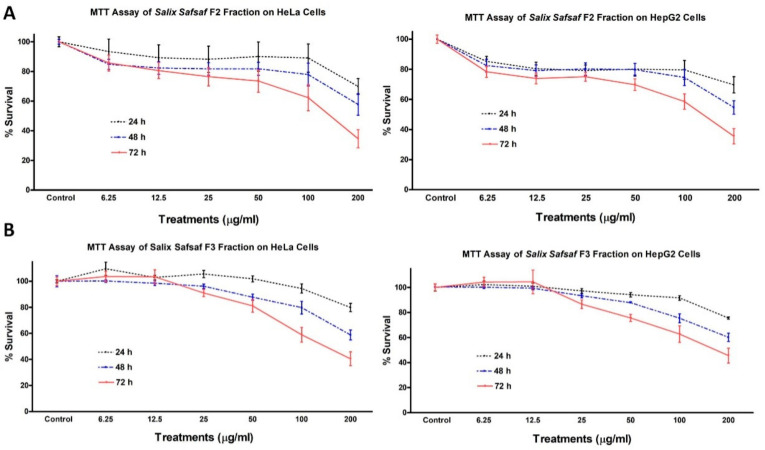
Effect of *Salix safsaf* extracts on cervical and liver cancer cells. HeLa and HepG2 cells were treated with indicated concentrations of *S. safsaf* fractions 2 and 3 (panels (**A**) and (**B**), respectively) or DMSO (solvent control) for 24, 48 or 72 h. Cell viability was determined by MTT assay as indicated in Materials and Methods. At the end of the assay, the absorbance at 549 nm was read on a microplate reader. Significant differences between treatments and control were analyzed by ANOVA followed by *t*-test.

**Figure 3:**
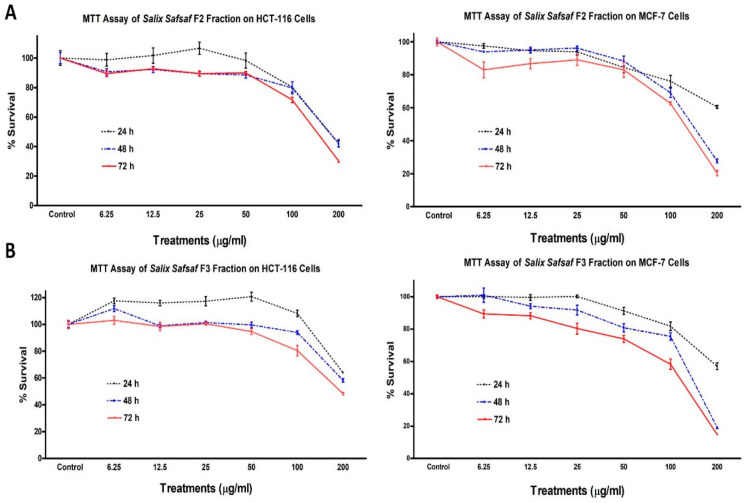
Effect of *Salix safsaf* extracts on colon and breast cancer cells. HCT-116 and MCF-7 cells were treated with indicated concentrations of *S. safsaf* fractions 2 and 3 (panels (**A**) and (**B**), respectively) or DMSO (solvent control) for 24, 48 or 72 h. Cell viability was determined by MTT assay as indicated in Materials and Methods. At the end of the assay, the absorbance at 549 nm was read on a microplate reader. Significant differences between treatments and control were analyzed by ANOVA followed by *t*-test.

Only two out of the seven fractions and extracts generated (Figure 1), F2 (CHCl_3_-soluble fraction) and F3 (EtOAc-soluble fraction), exhibited concentration and time-dependent anti-proliferative potency sufficient to measure (Figure 2 and Figure 3).

Potencies varied among the cancerous cell lines tested (Table 1). Since aqueous and ethanolic extracts of willow leaf have been shown previously to exhibit potent anti-leukemic activities [14,15], and F2 and F3 were the only fractions/extracts that exhibited significant anti-proliferative activity in the one-dose pre-screening further investigations were focused on F2 and F3. Concentrations causing 50% inhibition of growth of cells (IC_50_) were calculated by trendline equation, as previously described [16]. After 72 h treatment, IC_50_ for F2 and F3 gainst MCF-7 were 128.1 and 111.74 μg/mL, for HCT-116 were 151.49 and 195.56, for HeLa were 141.55 and 156.23, for HepG2 were 136.74 and 172.39, respectively (Table 1).

Therefore, MCF-7 was selected for the subsequent mechanistic studies as it is the most sensitive cancer cell line to the cytotoxicity of F2 and F3 fractions. Neither fraction had measurable effects on non-transformed skin fibroblast (BJ-1) and breast fibroblast (MCF-12) cells. Anti-proliferative activities of F2 and F3 against BJ-1 cells were 11.34 ± 3.41% and 6.12 ± 0.72%, whereas those of MCF-12 were 12.53 ± 2.71% and 7.61 ± 1.24% (data not shown), respectively, which indicated that both CHCl_3_- (F2) and EtOAc- (F3) soluble fractions exhibited selective cytotoxic potencies toward the four cancer cells, but not noncancerous BJ-1 and MCF-12 cells.

### 3.2. Identification of Phenolic Compounds Present in Chloroform (F2) and Ethyl Acetate (F3) Soluble Fractions of Willow Leaves

Based on the anti-proliferative potencies exhibited by F2 and F3 against the five selected cancerous cells, those fractions were subsequently subjected to full-scan (untargeted), Orbitrap mass spectrometric (MS) analysis to identify putative structures of some of the most abundant compounds present (Table 2 and Table 3).

**Figure 4:**
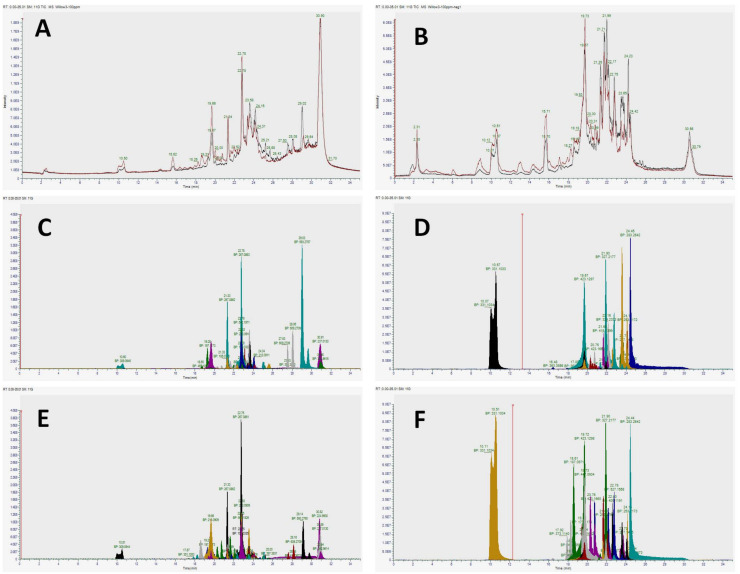
Total and extracted ion chromatograms generated by Orbitrap high resolution mass spectrometry (HRMS) analyses run in both positive and negative modes for the identification of secondary metabolites in *Salix safsaf* CHCl_3_- (F2) and EtOAc- (F3) soluble fractions. Superimposed total ion chromatograms of chloroform (black trace) and ethyl acetate (red trace) extracts run in positive (**A**) and negative (**B**) modes. (**C**,**E**) represent extracted ion chromatograms of the major positive mode features in 100 ppm for F2 and F3, respectively, identified by Compound Discoverer based on the feature filters described in the methods. Similarly, (**D**,**F**) represent extracted ion chromatograms of the major negative mode features in 100 ppm for F2 and F3 fractions, respectively. Peak colors (**C**–**F**) are not comparable across chromatograms. Only retention time and base peak (BP) should be compared when matching peaks.

Molecular formulae determined from accurate masses in both positive and negative ionization modes [M + H]^+^/[M − H]^−^, retention times (Rt, min) and abundancies (based on peak areas for compounds) are reported for each organic fraction. Unique features based on filtering settings with abundances according to minimum peak areas of ≥1 × 10^5^ within the 10 ppm/100 ppm ratio threshold are reported (Table 2 and Table 3). Accurate masses in both positive and negative ionization modes [M + H]^+^/[M − H]^−^, retention times (Rt, min) and abundancies (based on peak areas for compounds) are reported for each putative compound identification. Total ion chromatograms of F2 (black trace) and F3 (red trace) run in both positive and negative modes were quasi identical (Figure 4A–F).

Major classes of identified compounds were phenolic and flavonoid in nature that belong to the large family of secondary plant metabolites. Compounds tentatively identified in these classes included phenolic derivatives (catechin, vanillin, syringic acid, catechol, salicin), flavonoid aglycones (tangeritin, apigenin, isorhamnetin), flavonoid glycosides (quercetin, isoquercitin, rutin), and purine derivatives and cytokinins (trans-zeatin, 2-aminopurine, isopentenyladenosine, olomoucine, 6-methoxypurine, 2,6-diaminopurine, 6-anilinopurine, kinetin) (Table 2 and Table 3). According to peak areas, the predominant subclasses of compounds observed were catechols and purine derivatives.

### 3.3. Organic Fractions F2 and F3 of Willow Leaves Induce Apoptosis in MCF-7 Cells

**Figure 5:**
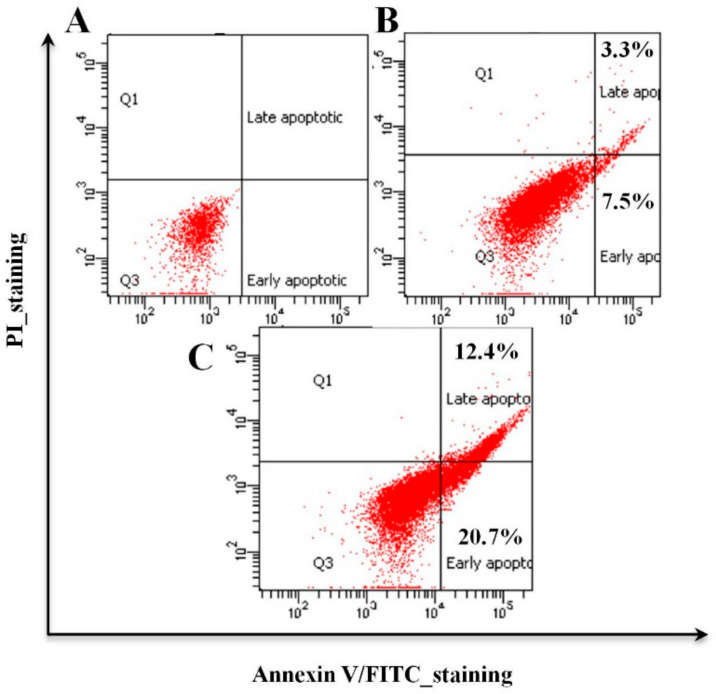
F2- and F3-mediated apoptosis induction in MCF-7 cells grown in 24-well plate 72 h post-treatment. MCF-7 cells were treated with IC_50_ concentrations of F2 (128.1 µg/mL) and F3 (111.72 µg/mL) for 72 h. Fluorescence-activated cell sorter analysis (FACS) was used in which a minimum of 20,000 events were acquired and analyzed following a forward scatter (FSC) and side scatter (SSC) gating strategy to identify the cells of interest and unstained cells for quadrant setup. Images represent: (**A**) FITC-Annexin V/PI double staining of untreated control cells (DMSO 0.1%), (**B**) cells treated with F2 exhibiting early apoptotic (7.5%) and late apoptotic (3.3%) changes, and (**C**) cells treated with F3 exhibiting early apoptotic (20.7%) and late apoptotic (12.4%) changes.

Cytotoxicity values of F3 against MCF-7 were 1.75-, 1.48- and 1.54-fold more potent compared to its effect on HCT-116, HeLa or HepG2 cells, whereas F2 exhibited a cytotoxic potency against MCF-7 cells that was 1.18 times that observed against HCT-116 (Table 1). To assess whether the observed growth inhibitory effect of the F2 and F3 originated from apoptosis, after exposure of MCF-7 cells to IC_50_ concentrations of F2 and F3 for 72 h, changes to the plasma membrane and/or loss of its integrity, which usually accompany early and late stages of cell death stemming from apoptotic or necrotic processes, respectively, were determined. The specific endpoint, investigated by use of FITC Annexin V in conjunction with the vital dye propidium iodide (PI) through FACS analysis (Figure 5A–C), resulted in early (PI negative, FITC/Annexin V positive) and late (PI positive, FITC/Annexin V positive) apoptosis-driven changes in membranes (Figure 5A–C). While MCF-7 cells treated with F2 exhibited 7.5% and 3.3% of early and late apoptotic changes, they exhibited 20.7% and 12.4% when treated with F3, respectively (Figure 5A–C). Collectively, MTT assay and apoptosis results clearly indicate that F3 exerts more potent anti-proliferative and pro-apoptotic activities against MCF-7 breast cancer cells.

### 3.4. Cyto-Protective Effects of Ethyl Acetate Fraction (F3) of Willow Leaves against Oxidative Stress in H_2_O_2_-Exposed WISH Cells

**Figure 6:**
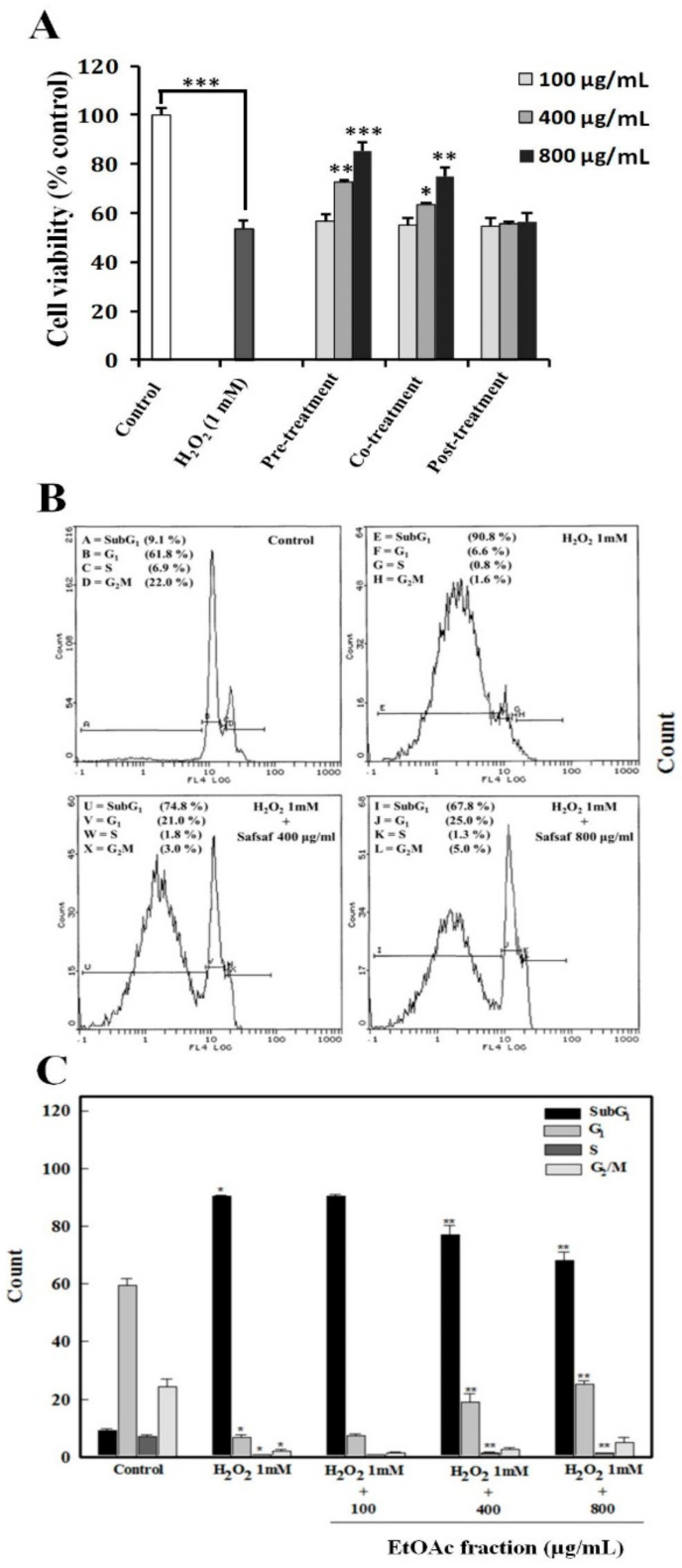
Chemo-preventive effects of willow leaf EtOAc fraction (F3) on H_2_O_2_-induced apoptosis in WISH cells. (**A**) Protective potential of F3 in WISH cells exposed to 1.0 mM hydrogen peroxide (H_2_O_2_) for 24 h. Three treatment schemes were tested: pre-treatment, co-treatment and post-treatment with three doses of 100, 400 and 800 µg/mL. Values are mean ± SD of three independent experiments. * *p* < 0.05, ** *p* < 0.01, *** *p* < 0.001. (**B**) A representative flow cytometric image from single experiment exhibiting changes in the progression of normal cell cycle in untreated control, H_2_O_2_-exposed and WISH cells pre-treated with F3 (400 and 800 µg/mL) plus H_2_O_2_ after 24 h. (**C**) Histogram depicting G1, S and G2/M in each micrograph representing the percentage of cells present in normal phases of cell cycle, whereas SubG1 represents percentage of cells that have undergone apoptosis/necrosis. Each histogram in **C** represents mean ± S.D. Values of different phases of cell cycle obtained from three independent experiments. Control: represents the solvent control (DMSO 0.1%); * *p* < 0.001 as compared to solvent control; ** *p* < 0.001 as compared to H_2_O_2_ (1 mM)-treated cells using one-way ANOVA (Man-Whitney U).

Antioxidant and chemo-protective potencies of F3 against oxidative stress (OS) generated by 1.0 mM H_2_O_2_ in WISH cells were observed. Cell viability was significantly (*p* < 0.001) less after a 24 h exposure to 1.0 mM H_2_O_2_ relative to untreated basal control (Figure 6A).

Cell viability was significantly rescued from effects of H_2_O_2_ following pre-treatment for 24 h at the doses of 400 (*p* < 0.01) or 800 (*p* < 0.001) µg F3/mL. Similarly, EtOAc fraction (F3)-mediated protection was observed during co-treatment but with relatively lesser protection at all the tested concentrations. By contrast, all the tested post-treatment doses of F3 were ineffective in blocking the detrimental effect of H_2_O_2_ on cell viability (Figure 6A).

Exposure to 1.0 mM H_2_O_2_ exhibited significant induction of apoptosis/necrosis as evidenced by the appearance of 90.8% of cells in Sub G1 phase of cell cycle *viz-á-viz* 9.1% of cells in solvent control (Figure 6B). Pre-treatment of WISH cells with 400 and 800 µg/mL of F3 followed by 24 h exposure to H_2_O_2_ resulted in a 14.9% and 24.7% decrease in the SubG1 (*p* < 0.001) peak compared to the H_2_O_2_-treated cell, respectively (Figure 6B). The two greatest concentrations of 400 or 800 µg F3/mL caused a return of 18.9% and 25.3% of cells to normal G1 phase relative to only 6.8% in cells exposed to H_2_O_2_ alone (Figure 6C). However, the least dose of 100 µg F3/mL was unable to reverse effects of H_2_O_2_ on apoptosis/necrosis (Figure 6C).

### 3.5. Ethyl Acetate Fraction (F3) of Willow Leaves Does Not Elicit ROS Elevation in MCF-7 Cell Line

**Figure 7:**
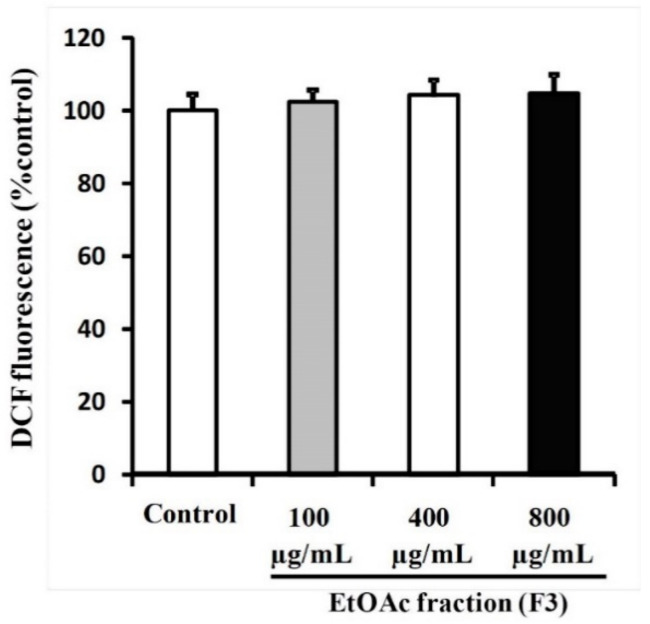
Investigation of F3-mediated ROS generation in MCF-7 cells. Apoptosis exhibited by MCF-7 cells treated with F3 does not involve elevated oxidative stress. MCF-7 cells were treated for 72 h with the 100, 400 and 800 µg/mL concentrations of F3. Control cells were treated with 0.1% DMSO. Cells were analyzed for ROS generation represented by monitoring the increase in DCF fluorescence after staining the cells with DCFDA.

Anti-proliferation effects and induction of apoptosis are often associated with the generation of reactive oxygen species (ROS), leading to DNA damage, cell cycle arrest and apoptosis. When analyses of cell cycle were conducted by FACS in MCF-7 cells treated with F3, production of ROS was not implicated for apoptosis caused by any of the three doses of 100, 400 or 800 µg F3/mL (Figure 7). No significant differences were observed between cells exposed to various concentrations of F3 or the solvent control (0.1% DMSO).

### 3.6. Apoptotic Marker Protein and mRNA Expression in F3-Treated MCF-7 Cells

**Figure 8:**
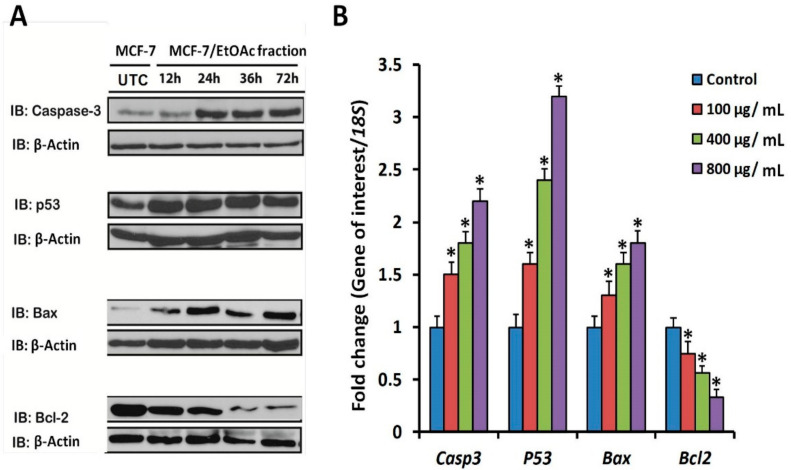
Analysis of apoptosis gene transcript and protein marker levels in MCF-7 cells treated with EtOAc fraction (F3) of willow leaves. (**A**) Immunoblotting of pro-apoptotic (Casp3, p53 and Bax) and anti-apoptotic (Bcl-2) protein markers after treatment with EtoAc F3 in comparison to untreated control (DMSO 0.1%). For immunoblots, 40 µg protein extracts were used, treated prior to harvest with 100 µg F3/mL and incubated for 12, 24, 48 and 72 h. Specific antibodies were used to detect p53, Bax, Caspase-3 and Bcl-2. Anti-β actin was used as loading control. (**B**) Profiling of mRNA transcript levels of key pro- (*Casp3*, *p53* and *Bax*) and anti-apoptotic (*Bcl-2*) genes in MCF-7 cells treated with three concentrations of EtOAc (F3) (100, 400 and 800 µg/mL). Gene expression levels were quantified after 72 h by RT-qPCR employing 18S as a housekeeping gene for normalization as detailed in the methods. Significant differences between the means of individual treatment and control were analyzed by a one-side Student’s *t*-test. Histograms represent mean expression level as fold change ± SEM for 3 technical and 2 biological replicas. Asterisk (*) denotes a significant difference at the probability level of *p* < 0.05 relative to solvent control (DMSO 0.1%).

It was hypothesized that F3 might affect expression of some markers of apoptosis at both the transcriptional and expression levels. Apoptosis is a process that is controlled by several signal transduction pathways including p53, Bax, caspase-3 and Bcl-2 proteins. Results of immunoblot analysis, using anti-p53, anti-Bax, anti-Caspase-3 and anti-Bcl-2 antibodies, indicated a time-dependent accumulation of p53, Bax and Caspase-3 protein markers in MCF-7 cells exposed to 100 µg/mL, relative to untreated ones (Figure 8A). By contrast, exposure to F3 significantly reduced the expression of the anti-apoptotic marker protein, Bcl-2. Similar patterns of expression were obtained with RTqPCR-based profiling of expressions of mRNAs of the same markers. Significant concentration-dependent increases in mRNA transcripts of the pro-apoptotic genes (*Casp3*, *p53* and *Bax*) were observed in cells exposed to 100, 400 or 800 µg F3/mL (Figure 8B). When MCF-7 cells were exposed to the greatest concentration of 800 µg F3/mL, fold changes in expressions of *Casp3*, *p53* and *Bax* mRNA reached maximum levels of 2.2 ± 0.12, 3.2 ± 0.1 and 1.8 ± 0.12, relative to the *18S* house keeping gene control, respectively (Figure 8B). However, the treatment resulted in significant reductions in the mRNA transcript levels of the anti-apoptotic marker gene *Bcl-2*, particularly in the two greatest concentrations of 400 and 800 µg F3/mL (Figure 8B).

## 4. Discussion

Plant-derived natural products (NPs) are valuable alternatives to synthetic chemo-therapeutic drugs. NPs are promising for developing novel chemical entities (NCEs) in the fight against cancer as they have been reported to be rich in bioactive, secondary metabolites such as phenolic and flavonoid compounds [9,22,23]. These ubiquitous compounds of phenolic nature have been shown to exhibit potent antineoplastic activities against several stages of carcinogenesis and associated inflammatory responses. NPs have been reported to be biologically friendly having wide margins of safety to normal cells, while exerting diverse pleiotropic effects on targeted cancerous cells, culminating in multiple favorable clinical outcomes [4,5,22,24].

Over the past two decades, our research group has published several reports that extended understanding of the antioxidant and anticancer properties of crude extracts of plants as well as their inherent active constituents and their modes-of-actions [14,15,17,19,25,26,27,28,29,30]. In this context, we were the first to report that aqueous and ethanolic crude extracts derived from young, emerging leaves of willow (*S. safsaf*) exhibit potent anti-leukemic properties [14,15]. The current study was designed to further explore the spectrum and modes-of-action of anticancer potential mediated by organic fractions and extracts of willow leaves, against four selected types of cancer cells, MCF-7, HCT-116, HeLa and HepG2. Of seven extracts/fractions generated (Figure 1), only chloroform-(CHCl_3_, F2) and ethyl acetate-(EtOAc, F3) soluble fractions of willow leaves exhibited potent concentration- and time-dependent anticancer activities (Figure 2 and Figure 3) as evidenced by IC_50_ values obtained from MTT cytotoxicity assay (Table 1). To our knowledge, this finding is the first report on anticancer properties of the organic-soluble fractions of *S. safsaf* in cancer types other than leukemia. It has been reported that ethanolic and ethyl acetate fractions derived from the bark of a related *Salix* species (*S. aegyptiaca*) have cytotoxicity against colorectal cancer cells, HCT-116 and HT-29 [31]. IC_50_ values for F3 were comparable to that reported for the EtOAc fraction from the bark of *S. aegyptiaca* against HCT-116 [31].

High resolution mass spectrometry (HRMS) has been reported to be one of the most powerful instrumental techniques for the structural elucidation of bioactive compounds in plant crude or fractionated mixtures [32]. This is because HRMS is not only characterized by its high sensitivity and versatility, high resolution and wide detection dynamic range, but also by its compatibility with chromatographic separation instruments [32]. Orbitrap-HRMS based untargeted analyses of CHCl_3_- and EtOAC-soluble fractions tentatively identified compounds from MS chromatograms in positive and negative modes as predominantly phenolic and flavonoid compounds, as well as the group of adenine-derived plant hormones, cytokinins (Figure 4A–F; Table 2 and Table 3). However, the identities of some features remained undetermined. In this context, it has been reported that for achieving unambiguous identification and confirmation of tentatively identified compounds with uncertain identity, inclusion of a reference standard is necessary. For further confirmation, isolation, purification and “de novo” identification by NMR is proposed to be essential [32]; however, this is beyond the scope of the current study. Structural elucidation of the Chinese herbal medicine *Scutellaria baicalensis* has been reported as a rapid alternative by use of UHPLC coupled with hybrid quadrupole orbitrap MS [33].

Accumulating evidence suggests that molecular mechanisms underlying the anticancer chemotherapeutic potential of plant-derived NPs are mediated via cell cycle arrest and up-regulation of pro-apoptotic markers [34]. It has been shown previously that observed antileukemic properties of aqueous and ethanolic extracts contain large amounts of salicin in young leaves, including apical meristem plus folded leaves [14]. In this study, it was found that salicin was predominantly present in the two extracts under investigation (Table 2 and Table 3), confirming the earlier results [14]. Results of the HRMS analysis also indicated that organic-soluble fractions were enriched with natural cytokines (CKs) and their derivatives. It was previously reported that Japanese willow trees exhibit in vitro anti-leukemic activities via inhibition of cell proliferation that is directly proportional to nitrogen content. Willow trees irrigated with 0.5 mM nitrogen for ten days showed significantly enhanced antileukemic potencies [35]. The association of nitrogen nutrition and CKs levels has been reported, where greater nitrogen fertilization results in a greater rate of delivery of CKs to leaves [36,37]. Moreover, CKs biosynthesis has been reported to be regulated by a nitrate-specific signal that controls expression of genes at a key step in the CKs biosynthetic pathway including adenosine phosphate-isopentenyltransferase (*IPT*) [37].

Results of FACS analysis, based on the Annexin V-FITC/PI assay, revealed that the observed potent anticancer activities of both F2 and F3 are mediated by early and late apoptotic events (Figure 5B,C) and that F3 was much more potent than F2. Hence, subsequent mechanistic studies were conducted using F3. In this context, evidence that the observed F3-mediated apoptosis proceeds via an ROS-independent pathway was presented (Figure 6), and that it possesses potent cytoprotective effects against oxidative stress, initiated by exposure to H_2_O_2_ (Figure 7). Data obtained from RT-qPCR and immunoblotting assays revealed that incubation of MCF-7 cells with F3 caused a significant induction of pro-apoptotic proteins and mRNA markers for Casp3, p53 and Bax, whereas it resulted in significant reduction in the mRNA and protein expression of the anti-apoptotic marker Bcl-2 (Figure 8A,B). Caspases are effector proteins that are vital for initiation and sustaining apoptosis. Upregulation of caspase 3 protein and mRNA transcripts (Figure 8A,B) is indicative of execution of the main intrinsic pathway of apoptosis; which is characterized by the collapse of the mitochondrial membrane with Bax-induced cytochrome c release, and activation of caspase 9 leading to the subsequent engagement of caspase 3 [17,19]. Up-regulation of Casp3, p53 and Bax, along with reduction of Bcl-2, expression in our study reveals that F3 induces the intrinsic pathway of apoptosis, where upregulation of p53 will stimulate expression of Bax, which, in turn, will induce cytochrome c release, followed by caspase-9 and -3 activation. Moreover, as Bcl-2 is known to inhibit cytochrome c release [17], in our study F3-induced Bcl-2 downregulation will facilitate unopposed Bax-induced cytochrome c release and subsequent apoptosis.

Results presented here are consistent with previous findings showing that methanolic extracts of Rosin (*Pix graeca*), the yellowish, translucent, brittle resin left after distilling the oil of turpentine from the crude oleoresin of pine, exhibit apoptosis-dependent anticancer activities against MCF-7 cells [19]. It has also been reported that the cytotoxicity exhibited by oxindole alkaloids extracted from the root bark of *Uncaria tomentosa* against acute lymphoblastic leukaemia cells is mediated through apoptosis [38]. Recently, organically-synthesized pyrazole-3,3′-oxindole analogues have been documented to exhibit apoptosis-dependent broad spectrum and specific anticancer activities against breast, colon and liver adenocarcinoma cells [39]. Similarly, findings reported here are consistent with previous reports that in vitro anti-leukemic potencies exhibited by aqueous extracts of emerging leaves from Japanese willow trees are mediated by an apoptosis-dependent process [35]. It has also been reported that active constituents of aloe (*Aloe vera*), namely: aloe-emodin, aloesin, barbaloin, octapeptide) exhibited anti-leukemic potencies, which are mediated by degradation of DNA and arrest of the cell cycle [26]. In line with our results, nobiletin, a similar polymethoxylated flavone derivative as that of tangeritin in *S. safsaf*, has been shown to exhibit anti-leukemic activities mediated by activation of MAPKs and caspases [40]. Furthermore, the dietary phenol o-coumaric acid has been reported to exhibit an anticancer activity against MCF-7 cells that is mediated via the up-regulation of pro-apoptotic (p53, caspase 3 and bax) and down-regulation of the anti-apoptotic marker Bcl-2, at both protein and mRNA levels [41], consistent with the findings reported here. O-coumaric acid also has been shown to cause cell cycle G1/S arrest taking place through a remarkable reduction in the mRNA and protein levels of cyclin D1 and cyclin dependent kinase-2 (CDK2) protein [41].

Results observed here are consistent with a previous study reporting that the inhibitory effect against medulloblastoma cells is due to thymoquinone (TQ), which is the main bioactive ingredient of black seed oil (*Nigella sativa*) from a flowering plant in the family Ranunculaceae, native to large regions around the eastern Mediterranean, including northern Africa. TQ mode-of-action has been proposed to be mediated by a caspase-dependent apoptotic mechanism [17]. Similar to TQ, syringic acid, a naturally occurring phenolic compound, has been reported to elicit an ROS-dependent cytotoxicity in hepatocellular carcinoma (HCC) and colorectal carcinoma (CRC) cells that is apoptosis-mediated [42,43]. In contrast, unlike that proposed for the activity of TQ [17], in this study, F3-mediated apoptosis was observed to be independent of ROS (Figure 7). The findings of the current study are, however, in agreement with inhibition of CRC cells by ethanolic extracts of bark (EEB) of the willow, *S. aegyptiaca* [31]. Similar to results obtained for F3, EEB cytotoxic activity has been proposed to proceed via an ROS-independent pathway [31]. Observed anticancer effects of F3 proceed via modulation of a p53-dependent apoptosis pathway, since both its protein and mRNA transcript levels were significantly up-regulated (Figure 6A,B). p53 is a protein that suppresses tumorogenesis, the expression and stability of which is negatively regulated by the PI3K/Akt pathway. In the event of cellular DNA damage or stress, p53 is engaged and subsequently translocated to the nucleus, where it initiates expressions of pro-apoptotic genes on mitochondrial membranes and activates effector caspases, which culminates in enhancement of apoptotic cell death [44]. Furthermore, EEB has been reported to induce Sub-G1 cell cycle arrest in CRC leading to significant increase in p21 protein levels [31]. In this context, the p21 protein is known to act as a negative regulator at the cell cycle transition point from G1 to S-phase. Thus, ablation of either p21 or Bax has been shown to block p53-dependent apoptosis induced by green tea polyphenol epigallocatechin-3-gallate [45]. Expression of p21 has been shown to be under direct transcriptional control of p53 [44]. It has been reported that p21 inhibition of the cdk2 activity is mdm2-dependent [46].

In this study mass spectral analyses revealed the presence of various phenolic and flavonoid compounds including catechol, CK-derivatives and salicin (Table 2 and Table 3). The characteristic antinociceptive and anti-inflammatory properties of willow bark have been proposed to stem from the ubiquitous combinatory effects of polyphenols inherently present in the extract, rather than because of the single constituent salicin [11,12,47]. These synergistic and additive effects have been described for several anticancer secondary metabolites and phytochemicals richly-present in fruits and vegetables [11,12]. Therefore, it is likely that the observed potent anticancer effects of F2 and F3 are not primarily attributed to salicin per se, but rather through its synergistic interaction with many polyphenolic compounds. Support of this suggestion comes from the finding that pure salicin, catechin and catechol do not exhibit any significant cytotoxicity on CRC cells, when administered individually. By contrast, the combination of catechin and catechol resulted in remarkable growth inhibitory effect on CRC cells [31]. It has been documented in vivo, however, that salicin exerts anticancer effects against Ehrlich ascites carcinoma (EAC) in mice, as evidenced by the reduction in tumor weight and volume, and elevation in carcinoembryonic antigen (CEA) marker level [48].

Apigenin is a natural flavonoid that has been recently reported to exhibit a dose-dependent anti-proliferative properties against CRC cells via the induction of apoptosis as evidenced by MAPK activation, PARP cleavage, suppression of the anti-apoptotic maker proteins Bcl-xL and Mcl-1 and inhibition of the phosphorylatic activation of signal transducer and activator of transcription 3 (STAT3) [49,50]. CKs and their various adenine analogues have been reported to be associated with potent in vitro anticancer activities [51,52]. Adenosine has been reported to exert dose- and time-dependent anti-proliferative activity against ovarian cancer cells (A2780 and SKOV3) that is mediated via apoptosis in an ROS-dependent mechanism [52]. The mechanism-of-action for purine compounds has been postulated to proceed through the activation of 5′-adenosine monophosphate-activated protein kinase (AMPK), a key cancer chemotherapeutic target. Thus, the purine analog ENERGI-F706 has been recently shown to exhibit significant AMPK-mediated anticancer activities against renal and HCC cells leading to cell cycle arrest and engagement of the apoptotic cascade [51,53]. Cytokinin ribosides (e.g., kinetin riboside, isopentenyladenosine and benzylaminopurine riboside), but not cytokinin, have been reported to result in significant in vitro anti-leukemic effects and the enhancement of apoptosis and its biochemical markers including reduction in intracellular ATP content, loss of mitochondrial membrane potential and generation of ROS [54]. Recently, the CK kinetin riboside (N6-furfuryladenosine) has been reported to selectively exhibit in vitro apoptosis-dependent anticancer activity [55].

Results reported here also revealed that F3 exhibits cytoprotective properties against oxidative stress (OS) mediated by the ROS molecule H_2_O_2_ in normal untransformed WISH cells (Figure 6A–C). Reports by our group and other researchers revealed that plant extracts and their active principles usually exhibit potent antioxidant capacities that accompany its anticancer potentials [26,28,29,30,45,56,57,58,59]. OS is a pathogenetic biochemical phenomenon that is frequently linked to numerous pathological conditions and diseases, including cardiovascular, neurodegenerative diseases, *diabetes mellitus*, cancer and aging [60]. OS is caused by an imbalance between oxidant and antioxidant capacities leading to detrimental and irreversible effects on plasma membranes and organelles, DNA damage and loss of enzymatic activities leading to numerous pathological conditions and genetic disorders [60]. The cytoprotective effect of plant-derived NPs is primarily associated with their polyphenolic compounds and their radical-scavenging capacities, thereby preventing inactivation of enzymes, loss of cellular integrity and insults to compartments [26,60]. Hydroxycinnamic acids are a class of natural aromatic acids or phenylpropanoids found in fruits and vegetables. These include coumaric acid, ferulic acid, sinapic acid, caffeic acid, chlorogenic acid and rosmarinic acid; all of which have been shown to exert cytoprotective effects associated with its potent antioxidant activity [61]. The anti-inflammatory properties of salicin have been attributed to its inhibitory effect on the inflammatory cytokine TNF-α [48], whereas its potent antioxidant activities against OS have been associated with promotion of the nuclear translocation of nuclear factor erythroid-2 (NRF2), which is a transcription factor that serves as a protective mechanism against ROS and OS that cause cellular damage [62]. Additionally, salicin exhibits a does-dependent anti-aging effect that is attributed to the inhibition of key senescence biomarkers, namely senescence-associated beta-galactosidase (SA-β-gal) and plasminogen activator inhibitor-1 (PAI-1) in human umbilical vein endothelial cells (HUVECs) [62].

Results presented here provided evidence that the CHCl_3_- and EtOAc-soluble fractions of willow leaves exhibit potent anticancer properties mediated by apoptosis via an ROS-independent mechanism. Data also revealed potent cytoprotective capacity of willow-leaf fractions in the H_2_O_2_-exposed non-malignant human amniotic epithelial (WISH) cells. Leaves of the willow, *S. Safsaf*, are a source of natural phenolic and flavonoid compounds with unparalleled and unique antioxidant and anticancer properties. The findings reported here warrant investigations of the active principles present in willow leaves in pre-clinical and clinical cancer trials.

## Figures and Tables

**Table 1:** IC_50_ values for willow leaves fractions/extracts against cancer cell lines.

Fraction/Extract	MCF-7	HCT-116	HeLa	HepG2
**F1**	NE	NE	NE	NE
**F2**	128.1	151.49	141.55	136.74
**F3**	111.74	195.56	156.23	172.39
**E1**	NE *	NE	NE	NE
**E2**	NE	NE	NE	NE
**E3**	NE	NE	NE	NE
**E4**	NE	NE	NE	NE

IC_50_ is the concentration (µg/mL) of a given fraction/extract that results in 50% inhibition of cellular growth. * NE indicates that no anti-proliferative effect was observed for the corresponding fraction/extract against the tested cancerous cell line. IC_50_ values were calculated using trendline equation [17]. F1, F2 and F3 are petroleum ether, CHCl_3_ and EtOAc sequential fractions, respectively. E1, E2, E3 and E4 are EtOH extract, 70% hydro-acetone extract, aqueous infusion and aqueous decoction, respectively.

**Table 2:** Tentative structures for identified compounds based on formulae from exact masses in positive mode, for CHCl3- (F2) and EtOAc- (F3) soluble fractions. The list is confined to the most predominant secondary metabolites shown in Figure 4C,E. Peak areas are for the 100 ppm fractions. Structure identification of this subset of peaks is based on literature evidence.

Name	Molecular Formula	Molecular Weight	[M + H]^+^	Rt (min)	Area F2	Area F3
**Catechol**	C_6_H_6_O_2_	110.0363	111.0441	15.63	5.69 × 10^6^	1.68 × 10^7^
**2-Aminopurine**	C_5_H_5_N_5_	135.0541	136.0619	17.17	4.57 × 10^6^	1.78 × 10^7^
**Vanillin**	C_8_H_8_O_3_	152.0469	153.0547	18.00	1.04 × 10^7^	1.34 × 10^7^
**Coumaric Acid**	C_9_H_8_O_3_	164.0471	165.0549	16.36	6.51 × 10^6^	4.70 × 10^7^
**Syringic Acid**	C_9_H_10_O_5_	198.0501	199.0579	10.90	NF	6.90 × 10^6^
**Trans-Zeatin**	C_10_H_13_N_5_O	219.1104	220.1182	7.30	NF	4.40 × 10^6^
**Adenosine**	C_10_H_13_N_5_O_4_	267.0964	268.1042	4.30	5.45 × 10^6^	3.47 × 10^7^
**Apigenin**	C_15_H_10_O_5_	270.0523	271.0602	22.17	1.27 × 10^7^	5.60 × 10^7^
**Salicin**	C_13_H_18_O_7_	286.1048	287.1126	20.01	NF	3.67 × 10^6^
**Olomoucine**	C_15_H_18_N_6_O	298.1539	299.1617	23.66	9.27 × 10^6^	NF
**Isopentenyladenosine**	C_15_H_21_N5O_4_	298.1540	299.1619	23.65	NF	2.13 × 10^7^
**Isorhamnetin**	C_16_H_12_O_7_	316.0584	317.0656	20.27	2.95 × 10^6^	2.95 × 10^6^
**Tangeritin**	C_20_H_20_O_7_	372.1209	373.1282	23.02	3.00 × 10^6^	4.05 × 10^6^
**Quercetin**	C_21_H_20_O_11_	448.1006	449.1084	19.65	NF	2.83 × 10^8^
**isoquercetin**	C_21_H_20_O_12_	464.0955	465.1028	18.88	1.49 × 10^7^	1.66 × 10^7^
**Rutin**	C_27_H_30_O_16_	610.1534	611.1612	18.30	1.08 × 10^6^	1.90 × 10^7^

NF—not found.

**Table 3:** Tentative structures for identified compounds based on formulae from exact masses in negative mode, for CHCl_3_- (F2) and EtOAc- (F3) soluble fractions. Figure 4D,F Peak areas are for the 100 ppm extracts. Structure identification of this subset of peaks is based on literature evidence.

Name	Molecular Formula	Molecular Weight	[M − H]^−^	Rt (min)	Area F2	Area F3
**Catechol**	C_6_H_6_O_2_	110.0373	109.0295	8.88	NF	1.24 × 10^9^
**6-Methoxypurine**	C_6_H_6_N_4_O	150.0547	149.0469	1.88	NF	2.67 × 10^6^
**2,6-Diaminopurine**	C_5_H_6_N_6_	150.0659	149.0581	21.82	NF	2.31 × 10^7^
**Coumaric Acid**	C_9_H_8_O_3_	164.0479	163.0401	12.9	NF	4.52 × 10^8^
**Gallic Acid**	C_7_H_6_O_5_	170.022	169.0142	2.12	NF	1.76 × 10^7^
**Syringic Acid**	C_9_H_10_O_5_	198.0533	197.0455	10.82	2.51 × 10^6^	6.91 × 10^6^
**6-Anilinopurine**	C_11_H_9_N_5_	211.0863	210.0785	12.64	NF	3.67 × 10^6^
**Kinetin**	C_10_H_9_N_5_O	215.0812	214.0734	2.28	NF	5.40 × 10^5^
**Trans-Zeatin**	C_10_H_13_N_5_O	219.1125	218.1047	2.33	1.50 × 10^6^	6.94 × 10^6^
**Benzyladenine**	C_12_H_11_N_5_	225.102	224.0942	19.95	NF	1.44 × 10^6^
**Adenosine**	C_10_H_13_N_5_O_4_	267.0973	266.0895	7.85	NF	9.08 × 10^5^
**Apigenin**	C_15_H_10_O_5_	270.0533	269.0455	22.33	1.84 × 10^7^	6.60 × 10^7^
**Salicin**	C_13_H_18_O_7_	286.1058	285.098	10.57	1.23 × 10^8^	1.68 × 10^8^
**Olomoucine**	C_15_H_18_N_6_O	298.1547	297.1469	17.35	4.33 × 10^5^	NF
**Geniposidic Acid**	C_16_H_22_O_10_	374.1213	373.114	18.36	5.38 × 10^6^	5.88 × 10^6^
**3,5,6,7,8,3′,4′-Heptemthoxyflavone**	C_22_H_24_O_9_	432.1419	431.1348	20.87	3.95 × 10^6^	5.21 × 10^6^
**Quercetin**	C_21_H_20_O_11_	448.1011	447.0933	19.72	2.16 × 10^7^	7.85 × 10^8^
**Rutin**	C_27_H_30_O_16_	610.1539	609.1461	18.5	4.78 × 10^6^	4.49 × 10^7^

NF—not found.
